# Analysis of the Gut Microbiota in the Old Order Amish and Its Relation to the Metabolic Syndrome

**DOI:** 10.1371/journal.pone.0043052

**Published:** 2012-08-15

**Authors:** Margaret L. Zupancic, Brandi L. Cantarel, Zhenqiu Liu, Elliott F. Drabek, Kathleen A. Ryan, Shana Cirimotich, Cheron Jones, Rob Knight, William A. Walters, Daniel Knights, Emmanuel F. Mongodin, Richard B. Horenstein, Braxton D. Mitchell, Nanette Steinle, Soren Snitker, Alan R. Shuldiner, Claire M. Fraser

**Affiliations:** 1 Institute for Genome Sciences, University of Maryland School of Medicine, Baltimore, Maryland, United States of America; 2 Department of Medicine, University of Maryland School of Medicine, Baltimore, Maryland, United States of America; 3 Department of Microbiology and Immunology, University of Maryland School of Medicine, Baltimore, Maryland, United States of America; 4 Department of Epidemiology and Preventive Medicine, University of Maryland School of Medicine, Baltimore, Maryland, United States of America; 5 Program in Personalized and Genomic Medicine, Division of Endocrinology, Diabetes, and Nutrition, Department of Medicine, University of Maryland School of Medicine, Baltimore, Maryland, United States of America; 6 Howard Hughes Medical Institute, Department of Chemistry and Biochemistry, University of Colorado at Boulder, Boulder, Colorado, United States of America; 7 Department of Molecular, Cellular and Developmental Biology, University of Colorado at Boulder, Boulder, Colorado, United States of America; 8 Veterans Administration Medical Center, Baltimore, Maryland, United States of America; The University of Texas Health Science Center (UTHSCSA), United States of AMerica

## Abstract

Obesity has been linked to the human gut microbiota; however, the contribution of gut bacterial species to the obese phenotype remains controversial because of conflicting results from studies in different populations. To explore the possible dysbiosis of gut microbiota in obesity and its metabolic complications, we studied men and women over a range of body mass indices from the Old Order Amish sect, a culturally homogeneous Caucasian population of Central European ancestry. We characterized the gut microbiota in 310 subjects by deep pyrosequencing of bar-coded PCR amplicons from the V1–V3 region of the 16S rRNA gene. Three communities of interacting bacteria were identified in the gut microbiota, analogous to previously identified gut enterotypes. Neither BMI nor any metabolic syndrome trait was associated with a particular gut community. Network analysis identified twenty-two bacterial species and four OTUs that were either positively or inversely correlated with metabolic syndrome traits, suggesting that certain members of the gut microbiota may play a role in these metabolic derangements.

## Introduction

Obesity, the accumulation of excess body fat has a negative impact on morbidity, mortality, and quality of life through its complications, which include cardiovascular disease, type 2 diabetes, osteoarthritis, and certain cancers [1]. Globally in step with the increase in industrialization, obesity has reached epidemic proportions such that overweight or obese humans now outnumber those suffering from malnutrition [2]. The etiology of obesity and its metabolic complications, including hyperlipidemia, hypertension, glucose intolerance and diabetes reflect the complex interactions of multiple genetic, behavioral, and environmental factors. Great inter-individual variation is apparent in the propensity toward obesity, the location where excess fat is deposited, and the extent to which this results in metabolic derangements and adverse health outcomes. Available treatments for obesity include lifestyle modification (diet and exercise), drugs, and bariatric surgery. With the possible exception of surgery, individuals often fail to maintain long term weight loss with these modalities. To offer better treatment and prevention modalities, deeper understanding of the etiology of obesity is needed. Novel lines of investigation implicate chronic inflammation [3] and the gut microbiome [4]–[6] in the development of obesity and its metabolic complications.

A 2005 publication by Ley et al. [4] provided evidence for a link between gut microbial ecology and obesity in a genetically homogeneous strain of leptin-deficient mice maintained in a highly controlled laboratory environment. More recent studies have suggested that microbes present in the human gut may also play a role in metabolism and adiposity; however, the results of these studies have been more variable, perhaps reflecting the complexity of human genetics and/or heterogeneity in lifestyle [7]. The emerging evidence that the microbiota may contribute in important ways to human health and disease has led us and others to hypothesize that both symbiotic and pathological relationships between gut microbes and their host may be key contributors to obesity and the metabolic complications of obesity [8]–[10]. We hypothesize that the gut microbiota influences host energy homeostasis, metabolism, and inflammation, and is an important determinant of obesity and its adverse health consequences.

To explore the possible dysbiosis of gut microbiota in obesity and its metabolic complications in humans, we studied Old Order Amish subjects from Lancaster County, Pennsylvania. The Amish are a genetically closed homogeneous Caucasian population of Central European ancestry ideal for such a study because of their high degree of social cohesiveness and common lifestyle [11], [12]. There is great uniformity of socioeconomic status and lifestyle among the Amish, and prescription medication usage is minimal, reducing the potentially confounding influences of variation in environmental exposures on complex traits. Extensive genealogies document a small number of founders and genetic analyses confirm less genetic heterogeneity relative to the general population. These attributes make the Amish a highly desirable population in which to study the composition of the gut microbiota, its heritability, and its relationship to obesity and metabolic complications. The specific questions we set out to address in this report were: (1) what are the major gut microbial subpopulations in this population and how does their distribution vary in obesity and with metabolic syndrome phenotypes? and (2) are socio-demographic and other factors associated with the major gut microbial subpopulations?

## Results

We enrolled a total of 310 adult subjects, of whom 112 were male and 198 were female. In this cohort, mean age and BMI was higher, and manifestations of the metabolic syndrome [13], were more common in women than in men (Table 1). We performed 16S rRNA multiplex pyrosequencing of V1–V3 amplicons using bar-coded primers on the 454 Titanium platform [14] to characterize the fecal microbiota. We obtained 10,357±3764 pyrosequencing reads per sample, with an average read length of 303 bp (Table S1). Reads were binned into individual samples based on the barcode sequence, and complementary phylogenetic and taxon-based analysis methods were used to compare 16S rRNA sequences across the fecal microbial communities (see Methods). In total, 203 genera were identified in the gut microbiota in the Amish; the 10 most abundant genera accounted for 67% of the reads (Figure S1). Seven species were each represented by more than 1% of the total sequence reads, including three species in the Bacteroidetes (*Prevotella copri, Bacteroides vulgatus* and *Bacteroides plebius*) and four species in the Firmicutes *(Faecalibacterium prauznitzii, Eubacterium rectale, Eubacterium biforme,* and *Roseburia faecis)* (Figure S1). The percent of reads for which a species-level taxonomic assignment could not be made averaged 47% across all the samples (range of 15% to 84%).

**Table 1:** Characteristics of subjects enrolled in this study.

	Men	Women
**N**	112	198
**Age (yr)**	46.0±12.7	49.5±13.4
**BMI (kg/m^2^)**	27.2±4.0 (19.3–42.3)	30.3±5.9 (16.7–51.1)
**Waist circumference (cm)**	95.6±11.2	90.3±12.3
**Systolic BP (mmHg)**	117.3±12.2	118.2±15.9
**Diastolic BP (mmHg)**	71.0±8.0	70.8±9.1
**Total cholesterol (mg/dl)**	208.7±42.5	214.4±50.2
**HDL-cholesterol (mg/dl)**	54.8±13.1	62.0±14.1
**LDL-cholesterol (mg/dl)**	138.6±39.0	134.7±45.6
**Triglycerides (mg/dl)**	76.4±45.5	88.5±54.0
**Glucose (mg/dl)**	87.4±8.0	87.3±11.2
**At Least 1 Metabolic Syndrome Trait (%)** *	26.8	38.9
**Blood Pressure (%)**	14.3	21.2
**HDL-C (%)**	8.9	18.7
**Triglycerides (%)**	3.6	11.6
**Glucose (%)**	7.1	10.6

* Metabolic syndrome traits were defined by NHLBI criteria: (1) fasting triglycerides >150 mg/dl (or on triglyceride lowering medication prescribed by a physician); (2) fasting HDL≤50 mg/dl for women or <40 mg/dl for men (or on HDL raising medication prescribed by a physician); (3) either or both systolic or diastolic blood pressure >130/85 mm Hg (or on anti-hypertension medication prescribed by a physician); (4) fasting glucose ≥100 mg/dl (or on anti-diabetes medication prescribed by a physician). Waist circumference was not included in our definitions because of the high correlation between waist circumference and BMI.

All but one of the twenty-five most abundant genera were present in at least 75% of all subjects, and these include members of the Firmicutes, Bacteroidetes, Tenericutes, Actinobacteria, and Proteobacteria (Figure S2). The prevalence of genera in the gut microbiota follows a bi-modal distribution, with a large peak above zero associated with many genera that are present in a small number of subjects and a second peak of 17 genera that are present in 95% or more of the 310 subjects (Figure S3). These 17 genera were deemed to comprise the core microbiota [15] in the Amish (Table 2) and represent members of the Firmicutes, Bacteroidetes and Tenericutes.

**Table 2:** Core gut microbiota at the genus level (present in ≥95% of subjects).

Firmicutes	Bacteroidetes	Tenericutes
*Clostridium*	*Bacteroides*	*Incertae sedis 32*
*Ruminococcus*	*Prevotella*	
*Faecalibacterium* *Subdoligranulum*		
*Oscillospira*		
*Coprococcus*		
*Blautia* *Roseburia* *Lachnospira* *Incertae sedis 121* *Lachnobacterium* *Dorea* *Eubacterium* *Streptococcus*		

Despite the substantial overlap in genera present in the gut microbiota of the 310 subjects, a significant amount of inter-individual variation was observed with respect to the relative abundance of both the predominant and rare genera (Figure S4). For example, the relative abundance of Bacteriodetes across all the samples ranges between 3 and 81%. Phyla-level phylogenetic binning of 16S rRNA data revealed a nominally significant correlation between the Bacteroidetes:Firmicutes ratio and age- and sex- adjusted BMI (r = 0.116; p = 0.04), but no significant correlation with metabolic syndrome traits [fasting glucose (r = 0.075; p = 0.19), systolic or diastolic blood pressure (r = 0.016; p = 0.78 and (r = 0.043; p = 0.45), respectively), fasting triglycerides (r = 0.078; p = 0.17), HDL-cholesterol (r = −0.052; p = 0.36); all adjusted for age and sex) Results from unweighted UniFrac analysis [16] also did not distinguish among the subjects based on BMI (Figure S5).

**Figure 1:**
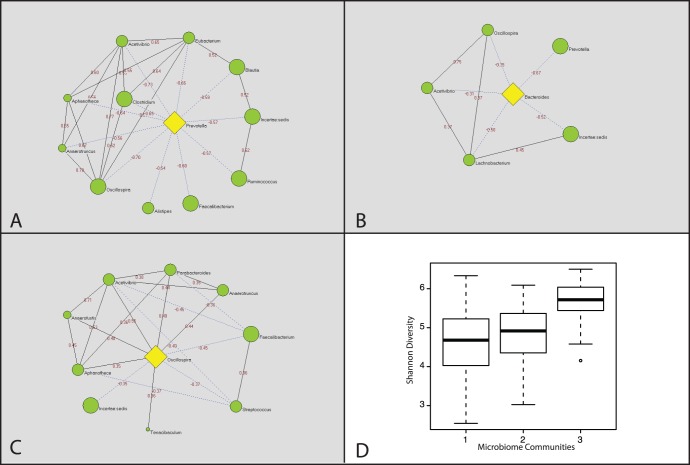
Bacterial networks in the human gut microbiota. Bacterial networks were identified based on statistically significant correlations among genera using the Louvain algorithm. Network I: *Prevotella*-dominated (A), Network II: *Bacteroides*-dominated (B) and Network III: *Oscillospira*-dominated (C) are illustrated, where the dominant bacterial genus is highlighted in yellow and other genera in green. The size of the circles represents the mean relative abundance of each genus in the OOA population. Solid lines represent positive correlations and dashed lines represent inverse correlations (all p<0.001). Numbers connecting microbes are the correlation coefficient. (D) Diversity in the three networks as measured by the Shannon Diversity metric.

**Figure 2:**
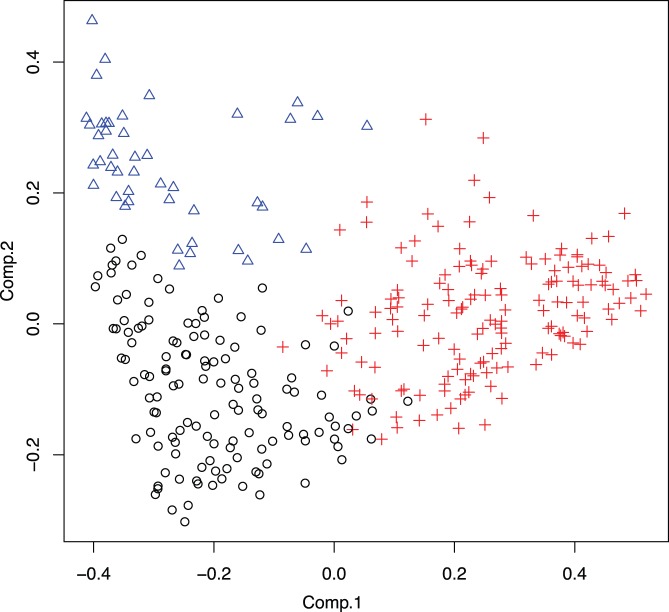
UniFrac Principle Co-Ordinates Analysis plot, showing each study sample positioned according to its first two principle coordinates. These are determined by the classical multidimensional scaling algorithm so that the Euclidean distance between points approximates the unweighted UniFrac distance between the OTU profiles of the corresponding samples, colored by Abundance of (A) Prevotella, (B) Bacteroides, and (C) Firmicutes.

Using a random matrix theory-based framework [17] we identified three networks of interacting bacteria in the human gut, that correlated with the three gut enterotypes recently reported by Arumugam et al. [18] (Figure 1A–C). In our dataset, these groups appear to represent a strong gradient effect between a few dominant taxa, rather than distinct clusters (Figure 2) because the silhouette width statistic [19] does not prefer three (mean silhouette width of 0.3) clusters over two (mean silhouette width of 0.34). Each subject was assigned membership to one of the bacterial groups based on the predominant genus present in his/her microbiota. The most prevalent group (I) in this cohort (47% of subjects: 55% male and 45% female) is dominated by *Prevotella*, the most abundant genus in the OOA gut microbiota. The least common group (II) in this cohort (14% of subjects: 23% male and 77% female), is *Bacteroides*-dominated. A Firmicutes-dominated group (III; 39% of subjects: 31% male and 69% female) is characterized by diverse Firmicutes genera, *Oscillospira* being the most abundant. Group III displays a statistically significant higher Shannon diversity index of both OTUs and named taxa than either network I or II (Figure 1D; p = 2.2×10^−16^ by Wilcoxon rank-sum test).

The three networks appear to reflect the main large-scale trends in gut microbial populations and suggest that gut bacterial community structures are driven by high abundance and high variability in populations of *Prevotella, Bacteroides*, or Firmicutes (with a degree of mutual exclusion between them). *Prevotella* and *Bacteroides* appear to co-exist at lower levels if the community is Firmicutes-dominated, but are nearly mutually exclusive when either is abundant in Bacteroidetes-dominated communities. Using community structure as a proxy, we examined the stability of the dominant taxa in 19 second samples from the subjects in this study 2–5 months following initial samples. The dominating taxa in 15 of the 19 subjects (79%) did not change (Figure S6). The communities became *Prevotella-*dominated in 4 subjects where a change was observed; in 3 cases first samples were collected in winter and the second samples were collected 3–5 months later in the spring. These changes may represent seasonal changes in diet that will need to be investigated further.

Our cohort of 310 subjects included 113 nuclear families having two or more members phenotyped. These nuclear families contributed a total of 54 spouse pairs, 76 sibling pairs, and 42 parent-offspring pairs. Community structure concordance rates tended to be lower for the spouse pairs (38.9%) than for the sibling (46.1%) and parent-offspring (52.4%) pairs (p = 0.32 for comparison of spouse pairs with the combined set of sibling and parent-offspring pairs). Larger sample sizes will be required to get better estimates of the relative contributions of relatedness and household effects to community structure.

We tested the association of groups (coded as a class variable with 3 levels) with each available clinical factor, while adjusting for age and sex (a 2 df test). Neither BMI nor any metabolic syndrome trait was associated with a particular group (Table 3). These data suggest that individual traits associated with obesity and its metabolic complications do not correlate with a major shift in the relative abundance of these two predominant phyla in the Amish, an observation reported in other cohorts [15], [20].

**Table 3:** Regression analyses between bacterial networks and metabolic phenotypes.

Variable	Network 1	Network 2	Network 3	p-value forNetwork Effect	Contrast	p-value
Age (yrs)	47.5±1.1	49.6±2.4	48.6±1.2	0.82		
Sex (% male)	44.5	23.3	30.6	0.01	Network 2 vs. Network 3Network 2 vs. Network 1Network 3 vs. Network 1	0.370.020.02
BMI (kg/m^2^)	29.1±0.5	29.3±0.8	29.4±0.5	0.79		
Waist (cm)	92.8±1.0	91.8±29.3	91.8±1.1	0.95		
HDL-cholesterol (mg/dl)	59.4±1.3	59.3±2.1	59.5±1.2	0.76		
Triglycerides (mg/dl)	82.5±4.3	96.5±9.8	81.8±4.1	0.39		
Glucose (mg/dl)	87.0±0.8	89.4±2.1	87.1±0.8	0.42		
Systolic BP (mm Hg)	117.6±1.1	117.5±2.2	118.3±1.4	0.88		
Diastolic BP (mm Hg)	71.1±0.7	71.3±1.3	70.4±0.9	0.69		
Reached Menopause (%)	39.8	19.3	41.0	0.72		
Has one or more metabolic syndrome traits (Yes/No) (%)	29.5	46.5	31.4	0.18		

All analyses adjusted for age and sex except analyses of age and sex, which were unadjusted. See Table 1 legend for definitions of metabolic syndrome traits.

To determine if gut community type was associated with occupation, we classified study subjects into occupational classes (farmers, tradesmen, farmer’s wives, teachers/shopkeepers, and unknown/retired) and tested the association of phenotype with each occupational class. In men, the occupation of farming was over-represented among those with the *Prevotella-*dominated network (71.4%) compared to those with either the *Bacteroidetes*-dominated (21.4%) or *Firmicutes*-dominated (36.5%) networks (p = 0.002; Table 4). The distribution of networks did not differ significantly within any other occupational class in men or with any occupational class in women (Table 4).

**Table 4:** Distribution of subjects in each microbiota network, according to occupational class.

	Men				Women			
	Network 1*Prevotella*(n = 65)	Network 2*Bacteroides*(n = 10)	Network 3Firmicutes(n = 37)	Age adj.p-value*	Network 1*Prevotella*(n = 81)	Network 2*Bacteroides*(n = 33)	Network 3Firmicutes(n = 84)	Age adj.p-value
Farmers	29 (44.6)	0 (0.0)	15 (40.5)	0.78	0 (0.0)	0 (0.0)	0 (0.0)	–
Tradesmen	22 (33.8)	5 (50.0)	16 (43.2)	0.51	0 (0.0)	0 (0.0)	0 (0.0)	–
Farmer’s wives	0 (0.0)	0 (0.0)	0 (0.0)	–	11 (13.6)	1 (3.0)	8 (9.5)	0.25
Teachers/shopkeepers	12 (18.4)	3 (30.0)	4 (10.8)	0.32	69 (85.2)	29 (87.9)	73 (86.9)	0.92
Unknown/retired	2 (3.1)	2 (20.0)	2 (5.4)	0.09	1 (1.2)	3 (9.1)	3 (3.6)	0.13

* p-value for test of association of occupational class with microbiota network.

**Figure 3:**
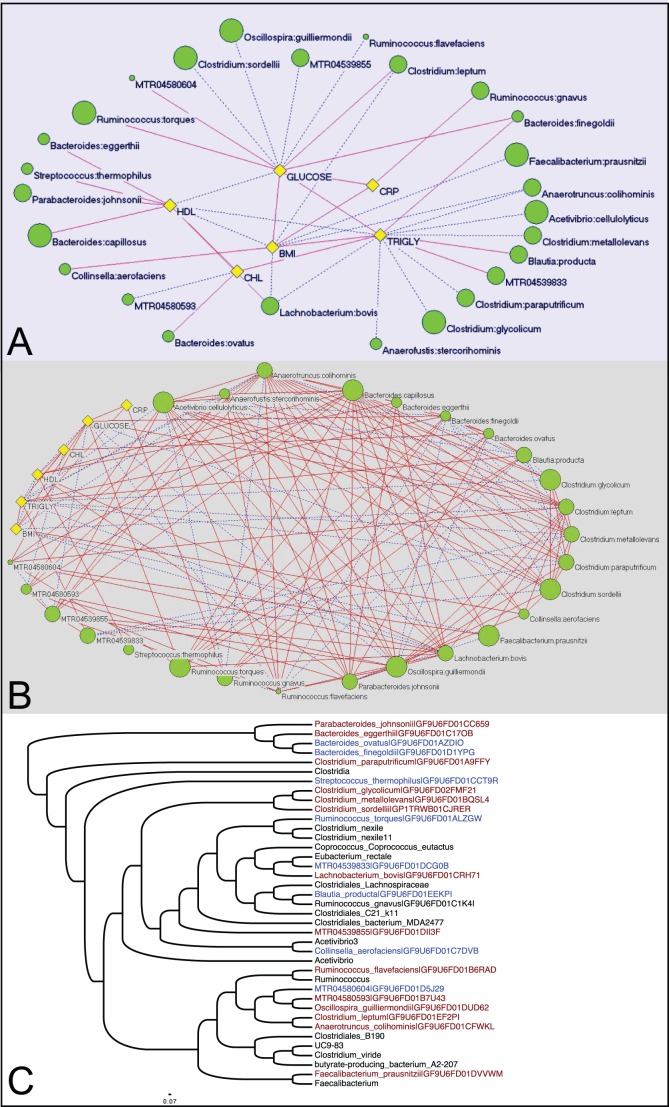
Bacterial species and OTUs correlated with metabolic syndrome phenotype. (A) Known species and Operational Taxonomic Units (OTUs) (green circles) linked to metabolic syndrome traits (yellow diamonds), illustrating statistically significant correlation coefficients using the Louvain algorithm. The size of the circles represents the mean relative abundance in the Amish cohort studied. Numbers connecting microbes are the correlation coefficient (p<0.001 for all). Solid lines represent positive correlations and dashed lines represent inverse correlations. (B) The same network as shown in panel A, but also including the statistically significant associations between bacterial taxa. (C) Phylogenetic tree of 16S rRNA sequences from the bacterial taxa in this network using the R implementation of DNADIST and FASTME. OTUs and known species that are inversely correlated with metabolic syndrome traits are colored in red and that are positively correlated with metabolic syndrome traits are colored in blue (p<0.001 for all).

The availability of extensive clinical data from the OOA cohort allowed us to evaluate the potential role of the microbiota (both species and OTUs) in obesity and its metabolic derangements using network analysis, independent of enterotype. Twenty-two species of bacteria from the phyla Bacteroidetes, Firmicutes, and Actinobacteria, and four OTUs from the order Clostridiales, displayed both positive and inverse correlations with BMI, serum triglycerides, HDL cholesterol, total cholesterol, fasting glucose levels and C-reactive protein (Figure 3A) and with each other (Figure 3B). These bacteria differ in both abundance and prevalence. Collectively, this group of species and OTUs represent between 0.03 and 31% of the total 16S rRNA sequence reads in the 310 subjects. Phylogenetic analysis revealed that the majority of these OTUs, along with the majority of known species associated with clinical phenotype are members of the order Clostridiales (Figure 3C). While a majority of the correlations observed were between one metabolic trait and one taxon, *Lachnobacterium bovis* and *Anaerotruncus colihominis* were inversely correlated with both high BMI and elevated serum triglycerides. Although these two species of gut bacteria are known to produce short chain fatty acids as end-products of metabolism, a more thorough understanding of the potential impact of these species on host metabolism awaits further studies that will be facilitated by completion of ongoing reference genome sequencing projects for gut-associated bacterial species [21].

In a subset of 32 older (≥59 years of age) obese subjects with more severe manifestations of the metabolic syndrome there was an increase in the relative abundance of three gram-negative bacterial genera, *Fusobacterium*, J2–29, and *Tenacibaculum*, as compared to younger subjects (Table S2). It is not possible to determine whether these represent age- or disease-related changes in the gut microbiota [26]; however, the increase in the abundance of *Fusobacterium* is of interest in light of previous findings that periodontal *Fusobacterium* species have been associated with inflammation, atherosclerosis [27] and colon cancer [28].

## Discussion

The prevalence of obesity has dramatically increased around the world over the last twenty years, in large part linked to the Western life-style. Many lines of evidence point to a complex etiology for obesity that includes both genetic and environmental factors. Obesity is associated with a panoply of co-morbidities including hypertension, dyslipidemia, insulin resistance, and diabetes, collectively known as the metabolic syndrome, that increase the risk of cardiovascular disease [1]. While numerous obesity-related changes in human physiology have been described, the gut microbiota has also been implicated in obesity, perhaps by influencing energy homeostasis, host signaling, insulin resistance, gut permeability, and inflammation and the innate immune response.

The present study used sequencing of 16S rRNA amplicons to characterize the gut microbiota in a metabolically well-characterized cohort of 310 Amish subjects over a range of BMI. The Amish are an excellent population to study the relationship between gut microbiota and metabolic traits because of their relatively homogeneous lifestyle. Furthermore, we believe that knowledge gained from the Amish is relevant to the general population because the clinical characteristics of obesity and its related complications in the Amish are indistinguishable from that in the general Caucasian population [22], and the Amish gene pool originates in Central Europe and thus host genetic findings in the Amish are likely to represent a subset of those present in the general Caucasian population.

Our analyses revealed several novel insights into the structure and role of the gut microbiota in obesity and the metabolic syndrome. First, we identified three groups of bacterial species, each of which include dominant organisms in the three enterotypes previously identified in a small set of European subjects [18]. These bacterial groups likely define an interactive functional community, where some members play redundant functional roles. Approximately 50% of the subjects enrolled in this study were members of nuclear families allowing us to compare concordance rates of community structure groups between related and unrelated individuals; however, these results did not provide any evidence that these structure groups aggregated within families. Interestingly, there was a correlation between occupation and community structure with farmers more likely than other occupations to harbor the *Prevotella*-dominated microbiota. To the extent that Amish famers come in closer contact with livestock than Amish in other occupations, this observation is intriguing in that the gut microbiota of various livestock species has been reported to contain a high relative abundance of the xylanolytic bacterial species *Prevotella* [23]–[25], although this result is also consistent with the interpretation that the ‘over-representation’ of the *Prevotella*-dominated microbiota is really due to an under-representation of the *Bacteroidetes* and *Firmicutes*-dominated phyla. Regardless, this leaves open the speculation that the environment may in some situations play an important role in modulating community composition, and may even suggest that transmission of gut microbes may occur across host species. This possibility is highly speculative, but suggests a potential avenue for future follow-up.

We hypothesized that we would identify differences in the gut microbiota that would be associated with body weight and/or features of the metabolic syndrome in this cohort. Neither B/F ratio nor community structure was associated with BMI or metabolic syndrome traits. Further investigation using network analysis identified 22 bacterial species and 4 OTUs that represent between 0.03 and 31% of the total sequence reads that were statistically significantly correlated with BMI and several features of the metabolic syndrome. Approximately half of these species are members of the core gut microbiota in the Amish and members of the Bacteroidetes and Firmicutes phyla.

In summary, we have carried out a survey of the gut microbiota and its relationship to obesity and metabolic syndrome in the OOA, a population of common genetic background and similar lifestyle. Our results have identified a subset of bacterial taxa that are linked to metabolic syndrome traits; although the cross-sectional nature of this study makes it difficult to infer cause and effect with these data alone. Follow-on longitudinal studies can begin to address whether specific gut bacterial taxa a play a causal role in the predisposition to or development of the metabolic syndrome, as well as the utility of interventions that modulate the composition of the gut microbiota to mitigate the risk of cardiovascular complications associated with metabolic syndrome.

## Materials and Methods

### Study Population and Sample Collection

Our study population consisted of Old Order Amish adults from Lancaster County, Pennsylvania. The Amish sect originated in Berne, Switzerland in 1693 as an ultraconservative wing of the Mennonite movement [11], [12]. Over approximately 50 years, beginning in 1727, a small number of Amish immigrated to eastern Pennsylvania. The Amish in Lancaster County have expanded to over 30,000 today. Our analyses indicate the Lancaster Amish population descended from a total of 275 founders, 76 of whom (31 men and 45 women) account for over 95% of the average founder contribution [29].

All recruitment was performed between April 2008 and September 2010. Eligibility criteria included the following: of Amish descent; age between 20 and 80 years. We recruited subjects over a wide range of BMIs. Exclusion criteria included the following: currently pregnant or have been pregnant in the last 6 months; antibiotic treatment within the prior 6 months; currently taking a medication (e.g., antibiotic, anti-inflammatory agents, glucocorticoids or other immune modulating medications), unwilling to discontinue vitamin or supplements, including probiotics, potentially affecting the gut microbiome (vitamins/supplements and medications that were judged to possibly affect the gut microbiome were discontinued for at least 14 days prior to stool collection); renal insufficiency (serum creatinine >2 mg/dl); hematocrit <32%; uncontrolled thyroid disease (TSH <0.4 or >5.5 mIU/; co-existing malignancy; history of intestinal surgery (except appendectomy or cholecystectomy); history of inflammatory bowel disease, celiac disease, lactose intolerance, chronic pancreatitis or other malabsorption disorder.

Recruitment was performed during an initial home visit by a field team consisting of a nurse and an Amish Liaison. A screening questionnaire, height, weight, and hip measurement, and blood tests (comprehensive metabolic panel, complete blood count, thyroid stimulating hormone (TSH), celiac screen; Quest Diagnostics, Inc, Horsham, PA) were obtained to rule out exclusions (see below). Eligible and consenting volunteers were provided with a stool sample collection kit and instructions for collection (see below). A follow-up home visit was conducted after an overnight fast to obtain additional information through questionnaires (such as medical and family history, food frequency), blood pressure measurements, and collection of blood, urine, and fecal samples.

All procedures were performed by trained personnel using standard operating procedures following the guidelines of the University of Maryland and the Amish Research Clinic [12]. Height and weight were measured by trained nurses in subjects without shoes and in light clothing using a stadiometer and calibrated scale. Body mass index was calculated as weight in kilograms divided by height in meters squared. Waist circumference was measured to the nearest 0.1 cm using an inelastic tape. Blood pressure was obtained manually with the subject in the sitting position after he or she had been sitting quietly for 5 minutes and the average of 3 measurements was used for analysis. Blood was drawn after a >8 hour fast and serum glucose, total cholesterol, HDL-cholesterol, and triglycerides, and C-reactive protein were assayed by Quest Diagnostics (Horsham, PA). LDL-cholesterol was calculated using Friedewald’s formula.

For collection of feces, subjects were instructed to collect a stool sample within 1 day of the scheduled home visit. The sample was collected in a disposable “nuns” hat, and a portion of the sample (approximately 1–2 g) was immediately dispersed in RNALater (QIAgen) and refrigerated overnight. RNALater-stabilized samples were then frozen at −80°C and transported on dry ice to the Institute for Genome Sciences for long-term storage. In a subset of 19 subjects, a second fecal sample was obtained 2 to 24 months later through a home visit. An interval history and follow-up anthropometry was also obtained at that home visit to assess any changes in health status or medication usage (including antibiotics). Protocols and procedures for this visit were similar to those in which the first fecal sample was obtained.

The Institutional Review Board at the University of Maryland School of Medicine approved the protocol and informed consent was obtained from all subjects.

### DNA Extraction

For DNA extraction, a 0.3 g stool aliquot was transferred to a DNA/RNA-free sterile tube, and 1 ml of phosphate-buffered saline was added to the sample. Cell lysis was initiated by adding 50 µL of lyzosyme (10 mg/mL) and 6 µL of mutanolysin (25,000 U/mL; Sigma- Aldrich). After a1 hour incubation at 37°C, each sample was further lysed by addition of 10 µl Proteinase K and 50 µl 10% SDS, followed by incubation at 55°C for 45 minutes. The samples were then disrupted by bead beating, which was performed in a FP120 FastPrep at 6.0 m/s for 40 sec using 0.1 mm silica spheres (QBiogen Lysis Matrix B). The resulting crude lysate was processed using the ZYMO Fecal DNA Kit (Zymogen) according to the manufacturer’s recommendations. Negative extraction controls, where stool samples were omitted, were performed to ensure the samples were not contaminated by exogenous bacterial DNA during the extraction process. The DNA concentrations in the samples were measured using the Quant-iT PicoGreen dsDNA assay kit from Molecular Probes (InVitrogen).

### Pyrosequencing of Barcoded 16S rRNA Gene Amplicons

Universal primers 27F and 338R were used for PCR amplification of the V1–V3 hypervariable regions of 16S rRNA genes. The 338R primer included a unique sequence tag to barcode each sample. The primers were as follows: 27F-5′-GCCTTGCCAGCCCGCTCAGTC**AGAGTTTGATCCTGGCTCAG**-3′ and 338R-5′-GCCTCCCTCGCGCCATCAGNNNNNNNNCAT**GCTGCCTCCCGTAGGAGT**-3′, where the underlined sequences are the 454 Life Sciences FLX sequencing primers B and A in 27F and 338R, respectively, and the bold letters denote the universal 16S rRNA primers 27F and 338R. The 8-bp barcode within primer 338R is denoted by 8 Ns. Using 96 barcoded 338R primers [30] the V1–V3 regions of 16S rRNA genes were amplified in 96-well microtiter plates using AmpliTaq Gold DNA polymerase (Applied Biosystems) and 50 ng of template DNA in a total reaction volume of 50 µL. Reactions were run in a PTC-100 thermal controller (MJ Research) using the following cycling parameters: 5 min of denaturation at 95°C, followed by 20 cycles of 30 s at 95°C (denaturing), 30 s at 56°C (annealing), and 90 s at 72°C (elongation), with a final extension at 72°C for 7 min. Negative controls without a template were included for each barcoded primer pair. The presence of amplicons was confirmed by gel electrophoresis on a 2% agarose gel and staining with SYBRGreen. PCR products were quantified using a GelDoc quantification system (BioRad) and the Quant-iT PicoGreen dsDNA assay. Equimolar amounts (100 ng) of the PCR amplicons were mixed in a single tube. Amplification primers and reaction buffer were removed from each sample using the AMPure Kit (Agencourt). The purified amplicon mixtures were sequenced by 454 FLX Titanium pyrosequencing using 454 Life Sciences primer A by the Genomics Resource Center at the Institute for Genome Sciences, University of Maryland School of Medicine, using protocols recommended by the manufacturer as amended by the Center.

### Sequence Analysis

Sequences were binned and trimmed, using the sample-specific barcode sequences, using mothur and the following criteria: (i) sequence length >199 bases, (ii) sequence length <501 bases, number of ambiguous bases = 0, exact barcode matching, 1 nucleotide mismatch in primer matching, and maximum homopolymer string of 8 bases [31]. Taxonomy assignments were done by kmer-based naive-Bayes classification via mothur classify.seqs applied to the GreenGenes [32] reference sequences and taxonomy, with a confidence cut-off of 0.5. Operational taxonomic units (OTUs) were determined using mothur by (i) alignment to the SILVA 16S rRNA database [33], (ii) clustering by bacterial family [31], and a distance matrix cutoff of 0.03. Jensen-Shannon divergence between genus-level frequency distributions was calculated for each pair of samples, and the square-root of this was used to perform hierarchical clustering with complete linkage via the R hclust command. The three top-level clades were then used as the three groups.

### Construction of Bacterial Networks

For each network, we transformed the 16S rRNA sequence reads into relative abundance, computed the Spearman rank correlation, and then constructed a genera network and identified a sub-network (module) with those genera that have a direct connection. The network was constructed with the cutoff p value of less than 0.001.

### Network Analysis with Integrated 16S rRNA Sequence and Clinical Data

To visualize the interconnectivities between species in the human gut microbiota and clinical phenotypes, we transformed the number of 16S rRNA sequence reads from each sample into relative percentages, and then computed the cross-correlation matrix between clinical metadata and sequence data with the Spearman rank correlation. The network was constructed with correlations that have a p-value of 0.005 or less. The sub-networks (modules) are identified with the Louvain algorithm [34].

### Phylogenetic Tree Construction

Representative 16S rRNA sequences assigned to the 13 species and 12 OTUs with correlations to MST, were aligned to the SILVA database of reference 16S rRNA genes [33] using MOTHUR (align.seq) and trimmed based on reference coordinates using screen.seqs/filter.seqs to include sequences that were atleast 200 bp over the V1–V3 region. Distances were calculated with the trimmed multiple sequence alignment using DNADIST [35] in the R APE package. A phylogenetic tree was constructed using FASTME [36], [37].

### Association of Major Networks with Demographic and Other Factors

We compared the distribution of the three major groups across a variety of factors including age, sex, occupation, season of feces collection, and metabolic syndrome-related phenotypic characteristics of the study subjects. We tested the association of bacterial groups with demographic and metabolic factors by regressing group, coded as a class variable with 3 levels, against each metabolic variable separately, and adjusting for age and sex. This was a 2 df test. Continuously distributed variables were compared across groups using analysis of variance. Whenever a significant association was detected, we then tested each pairwise contrast separately (e.g., group 1 vs groups 2 and 3; group 2 vs groups 1 and 3; and group 3 vs groups 1 and 2) to determine the relative contributions of each contrast to the difference. These analyses were run in SAS using the GLM procedure.

Because our sample of 310 individuals included 113 nuclear families, we were able to estimate the heritability of enterotype by comparing concordance rates for enterotype class between spouse pairs (who are unrelated) and sib-pairs and parent-offspring pairs (who share 50% of their genes in common). We estimated heritability as twice the difference in concordance rates between the first-degree relative pairs and the spouse pairs.

## Supporting Information

Figure S1 **Rank abundance of genera in the gut microbiota of the Amish.** Relative abundance of the top 100 species identified across all samples in this study.
(EPS)

Figure S2 **Prevalence of bacterial genera in the Amish.** The prevalence of a genus was calculated as the percent of subjects in which that genus is present. Genera are colored by percent mean relative abundance, 0–0.42 (blue), 0.42–1.6 (purple), 2.6–5.4 (navy) and 6.1–28 (red).
(EPS)

Figure S3 **Distribution of genera in the Amish.** Histogram of prevalences among all 203 genera observed in the Amish cohort studied. The peak on the left represents the many genera observed in a small number of samples, while the peak on the right represents the core microbiota, observed in 95% or more of the samples.
(EPS)

Figure S4 **Bacteroidetes and Firmicutes composition in the gut microbiota of 310 Old Order Amish subjects.** Bar plots show the fraction of total sequence reads assigned to the bacterial families Bacteroidaceae, Prevotellaceae, Lachnospiraceae, and Ruminococcaceae in lean women (FN), lean men (MN), overweight/obese women (FB) and men (MB) without, and overweight/obese women (FM) and men (MM) with metabolic syndrome traits.
(EPS)

Figure S5 **UniFrac distribution of OOA subjects based on BMI.** UniFrac Principle Co-Ordinates Analysis plot, showing each study sample positioned according to its first three principle coordinates. These are determined by the classical multidimensional scaling algorithm so that the Euclidean distance between points approximates the unweighted UniFrac distance between the OTU profiles of the corresponding samples. For this analysis, subjects were divided into 5 non-overlapping phenotype groups: Normal weight (BMI<25 kg/m^2^)(N = red, n = 73); overweight (BMI 25.0–29,9 kg/m^2^) with no features of the metabolic syndrome (OvB = yellow, n = 77); overweight with one or more features of the metabolic syndrome (OvM = green, n = 23), obese (BMI >30.0 kg/m^2^ ) with no features of the metabolic syndrome (OB = green, n = 59), and obese with one or more features of the metabolic syndrome (OM = violet, n = 77). Features of the metabolic syndrome were defined by NHLBI criteria: (1) fasting triglycerides >150 mg/dl (or on triglyceride lowering medication prescribed by a physician); (2) fasting HDL≤50 mg/dl for women or ≤40 mg/dl for men (or on HDL raising medication prescribed by a physician); (3) either or both systolic or diastolic blood pressure >130/85 mm Hg (or on anti-hypertension medication prescribed by a physician); (4) fasting glucose >100 mg/dl (or on anti-diabetes medication prescribed by a physician). Waist circumference was not included in our definitions because of the high correlation between waist circumference and BMI.
(EPS)

Figure S6 **Community profile comparison in samples from the same subject**. Principal Co-Ordinates Analysis plot, showing each study sample positioned according to its first two principle coordinates of the square root of the Jensen-Shannon divergence. Samples belonging to the same sample are denoted with the same symbol. Phenotype is indicated by specifically normal weight, BMI<25 kg/m^2^ (red); overweight and obese with no features of the metabolic syndrome, BMI >25.0 kg/m^2^ (purple) and overweight and obese with features of the metabolic syndrome, BMI >25.0 kg/m^2^ (blue).
(EPS)

Table S1 **Sequencing Statistics.**
(DOCX)

Table S2 **Regression analyses for phenotype clusters in the OOA.**
(DOCX)
