# Supplementary material for: Analysis of the Gut Microbiota in the Old Order Amish and Its Relation to the Metabolic Syndrome
Source: PLoS One. 2012 Aug 15;7(8):e43052. doi: 10.1371/journal.pone.0043052 (PMC3419686; doi:10.1371/journal.pone.0043052)
Supplement: Table S1 — Sequencing Statistics. (DOCX) [file pone.0043052.s007.docx]

**Supplementary Table 1.** Sequencing Statistics

| Weight | Phenotype | # of Visit 1 Samples | # of Visit 2 Samples | # of Visit 1 Reads (K) | # of Visit 2 Reads (K) | AVG Visit 1 Reads (K)/  Sample | AVG Visit 2 Reads (K)/  Sample | # of Visit 1 BP (M) | # of Visit 2 BP (M) | AVG V1 Read Length | AVG V2 Read Length |
| --- | --- | --- | --- | --- | --- | --- | --- | --- | --- | --- | --- |
| Normal |  | 75 | 5 | 855 | 63 | 11 | 13 | 260 | 20 | 305 | 310 |
| Overweight | No features* of metabolic syndrome | 68 | 0 | 638 | - | 9 | - | 183 | - | 287 | - |
|  | One or more features of metabolic syndrome | 22 | 1 | 219 | 16 | 10 | 16 | 73 | 5 | 333 | 321 |
| Obese | No features* of metabolic syndrome | 66 | 4 | 698 | 34 | 11 | 9 | 214 | 10 | 306 | 304 |
|  | One or more features of metabolic syndrome | 79 | 9 | 799 | 81 | 10 | 9 | 281 | 26 | 352 | 316 |
| Total |  | 310 | 19 | 3,209 | 196 | 10 | 12 | 1,012 | 61 | 317 | 313 |
