# Supplementary material for: Analysis of the Gut Microbiota in the Old Order Amish and Its Relation to the Metabolic Syndrome
Source: PLoS One. 2012 Aug 15;7(8):e43052. doi: 10.1371/journal.pone.0043052 (PMC3419686; doi:10.1371/journal.pone.0043052)
Supplement: Table S2 — Regression analyses for phenotype clusters in the OOA. (DOCX) [file pone.0043052.s008.docx]

| **Variable** | | Phenotype  Cluster 1 (P1) | | Phenotype  Cluster 2 (P2) | | Phenotype  Cluster 3 (P3) | | p-value for Network Effect | Contrast | p-value |
| --- | --- | --- | --- | --- | --- | --- | --- | --- | --- | --- |
|  | | Male | Female | Male | Female | Male | Female |  |  |  |
| **Age (yrs)** | | 43.1 ± 11.8 | 48.0 ± 13.7 | 45.6 ± 12.6 | 47.7 ± 12.1 | 59.3 ± 8.4 | 62.7 ± 10.3 | 3.60E-09 | P2 vs P3 | 1E-9 |
|  |  |  |  |  |  |  |  |  | P1vs P3 | 1.3E-8 |
| **Sex (% M)** | | 29.8 |  | 42 |  | 31.3 |  | 0.09 | P1 vs P2 | 0.035 |
|  |  |  |  |  |  |  |  |  | P2 vs P3 | 0.26 |
|  |  |  |  |  |  |  |  |  | P1 vs P3 | 0.87 |
| **BMI (kg/m^2^)** | | 24.5± 2.7 | 27.4±5.2 | 28.3 ± 3.7 | 32.2±5.3 | 30.1 ± 4.7 | 33.8±6.0 | 8.40E-13 | P1 vs P2 | 7.1E-11 |
|  |  |  |  |  |  |  |  |  | P2 vs P3 | 0.04 |
|  |  |  |  |  |  |  |  |  | P1 vs P3 | 6E-9 |
| **Waist (cm)** | | 87.9±7.7 | 84.8±11.6 | 98.6±10.3 | 94.0±11.2 | 104.0±12.8 | 96.4±10.6 | 8.73E-13 | P1 vs P2 | 2.4E-13 |
|  |  |  |  |  |  |  |  |  | P2 vs P3 | 0.61 |
|  |  |  |  |  |  |  |  |  | P1 vs P3 | 0.0001 |
| **HDL-cholesterol (mg/dl)** | | 66.9 ± 13.1 | 48.7± 12.0 | 48.5 ± 7.7 | 52.6± 8.1 | 53.5 ± 12.2 | 59± 15.3 | 4.00E-39 | P1 vs P2 | 9.1E-42 |
|  |  |  |  |  |  |  |  |  | P2 vs P3 | 6.4E-4 |
|  |  |  |  |  |  |  |  |  | P1 vs P3 | 3.5E-7 |
| **Triglycerides (mg/dl)** | | 54.1 ± 20.5 | 62.5± 26.2 | 88.5 ± 52.4 | 105.6± 60.1 | 77.5 ± 31.5 | 118.7± 64.6 | 4.10E-11 | P1 vs P2 | 4.1E-11 |
|  |  |  |  |  |  |  |  |  | P2 vs P3 | 0.5 |
|  |  |  |  |  |  |  |  |  | P1 vs P3 | 5.5E-10 |
| **Glucose (mg/dl)** | | 83.6± 7.8 | 83.3± 6.8 | 88.9± 7.1 | 89.8± 13.6 | 91.6± 9.4 | 92.6± 8.2 | 2.50E-08 | P1 vs P2 | 4E-7 |
|  |  |  |  |  |  |  |  |  | P2 vs P3 | 0.178 |
|  |  |  |  |  |  |  |  |  | P1 vs P3 | 9.2E-9 |
| **Systolic BP (mm Hg)** | | 111.1± 9.1 | 112.3± 12.1 | 116.8± 9.2 | 116.1± 10.5 | 142.3± 7.8 | 149.4± 11.8 | 2.10E-44 | P1 vs P2 | 0.00055 |
|  |  |  |  |  |  |  |  |  | P2 vs P3 | 1.1E-35 |
|  |  |  |  |  |  |  |  |  | P1 vs P3 | 1.2E-33 |
| **Diastolic BP (mm Hg)** | | 67.3± 4.7 | 68.0± 7.2 | 70.8± 7.2 | 70.4± 7.2 | 85.4± 7.0 | 83.0± 10.5 | 1.80E-22 | P1 vs P2 | 0.0015 |
|  |  |  |  |  |  |  |  |  | P2 vs P3 | 2.7E-15 |
|  |  |  |  |  |  |  |  |  | P1 vs P3 | 2.8E-21 |
| **LDL-cholesterol (mg/dl)** | | 154.1± 38.8 | 150.2± 53.9 | 129.0± 33.0 | 117.7± 32 | 145.8± 58.2 | 145.2± 34.3 | 5.00E-08 | P1 vs P2 | 1.6E-8 |
|  |  |  |  |  |  |  |  |  | P2 vs P3 | 7.5E-4 |
|  |  |  |  |  |  |  |  |  | P1 vs P3 | 0.5 |
| **CHL-cholesterol (mg/dl)** | | 231.8± 38.5 | 235.5± 54.4 | 195.2± 35.5 | 191.4± 36.7 | 214.8± 61.5 | 228.0± 41.8 | 1.70E-13 | P1 vs P2 | 3.4E-14 |
|  |  |  |  |  |  |  |  |  | P2 vs P3 | 5.4E-5 |
|  |  |  |  |  |  |  |  |  | P1 vs P3 | 0.3 |
| **MAP** | | 81.9± 5.3 | 82.8± 8.2 | 86.1± 6.7 | 85.7± 7.9 | 104.5± 5.2 | 105.3± 9.0 | 2.20E-38 | P1 vs P2 | 2.4E-4 |
|  |  |  |  |  |  |  |  |  | P2 vs P3 | 1.5E-28 |
|  |  |  |  |  |  |  |  |  | P1 vs P3 | 7.5E-32 |
| **CRP-**  **C-Reactive Protein** | | 2.03± 3.6 | 1.8±2.4 | 2.8± 6.6 | 3.0±4 | 6.7±13 | 2.2± 2 | 8.00E-02 | P1 vs P2 | 0.04 |
|  |  |  |  |  |  |  |  |  | P2 vs P3 | 0.5 |
|  |  |  |  |  |  |  |  |  | P1 vs P3 | 0.04 |
| **Reached**  **Menopause (%)** | | | 42.86% |  | 31.87% |  | 81.82% | 0.1 | P1 vs P2 | 0.17 |
|  |  |  |  |  |  |  |  |  | P2 vs P3 | 0.49 |
|  |  |  |  |  |  |  |  |  | P1 vs P3 | 0.17 |
| **One or more MetS traits (%)** | 8.33% | | 14.12% | 25.76% | 47.25% | 100.00% | 100.00% | 3E-18 | P1 vs P2 | 4.3E-07 |
|  |  | |  |  |  |  |  |  | P2 vs P3 | 0.002 |
|  |  | |  |  |  |  |  |  | P1 vs P3 | 2.4E-06 |

**Supplementary Table 2. Regression analyses for phenotype clusters in the OOA**
